# Native Arbuscular Mycorrhizal Fungi Characterization from Saline Lands in Arid Oases, Northwest China

**DOI:** 10.3390/jof6020080

**Published:** 2020-06-06

**Authors:** Erica Lumini, Jing Pan, Franco Magurno, Cuihua Huang, Valeria Bianciotto, Xian Xue, Raffaella Balestrini, Anna Tedeschi

**Affiliations:** 1 National Research Council of Italy, Institute for Sustainable Plant Protection, 10135 Turin, Italy; valeria.bianciotto@ipsp.cnr.it (V.B.); raffaella.balestrini@ipsp.cnr.it (R.B.); 2 Drylands Salinization Research Station, Key Laboratory of Desert and Desertification, Northwest Institute of Eco-Environment and Resources, Chinese Academy of Sciences, 320 West Donggang Road, Lanzhou 730000, China; panjing@lzb.ac.cn (J.P.); hch@lzb.ac.cn (C.H.); xianxue@lzb.ac.cn (X.X.); 3 Department of Botany and Nature Protection, Faculty of Biology and Environmental Protection, University of Silesia in Katowice, Jagiellońska 28, 40-032 Katowice, Poland; franco.magurno@gmail.com; 4 Centre of Mountain Environmental Technologies, 43-438 Brenna, Poland; 5 National Research Council of Italy, Institute for Agricultural and Forestry Systems in the Mediterranean, 80056 Ercolano, Italy; anna.tedeschi@cnr.it

**Keywords:** arbuscular mycorrhizal fungi (AMF), dry desert region, halophytes, soil biodiversity

## Abstract

Arbuscular mycorrhizal fungi (AMF) colonize land plants in almost every ecosystem, even in extreme conditions, such as saline soils. In the present work, we report the mycorrhizal capacity of rhizosphere soils collected in the dry desert region of the Minqin Oasis, located in the northwest of China (Gansu province), which is characterized by several halophytes. *Lycium* spp. and *Peganum nigellastrum* were used as trap plants in a greenhouse experiment to identify autochthonous AMF associated with the halophytes’ rhizospheres. Morphological observations showed the typical AMF structures inside roots. Twenty-six molecularly distinct AMF taxa were recovered from soil and root DNA. The taxonomical diversity mirrors the several AMF adapted to extreme environmental conditions, such as the saline soil of central China. Knowledge of the AMF associated with halophytes may contribute to select specific fungal isolates to set up agriculture strategies for protecting non-halophyte crop plants in saline soils.

## 1. Introduction

Worldwide, soil salinity is a significant threat encountered in all climates. Soils are rendered saline due to the deposition of salt either by natural (primary) or anthropogenic (secondary) processes [1]. Anthropogenic activities, such as poor drainage facilities, irrigation with brackish groundwater, continuous irrigation for long durations, improper management of water, and cultural methods in irrigated agriculture, combined with low rainfall, convert fertile arable land into salt-affected lands, and nearly 7% of agricultural land area is affected by salinity [2]. The Minqin Oasis, located in the northwest of China (Gansu province), is affected by salinity and desertification due to intense anthropogenic activities, and represents a suitable site for research on the impact of soil salinity. In the Minqin Oasis, 70% of farmland is affected by aeolian desertification and 85% is affected by different degrees of salinization, which causes harmful effects on crop production [3]. 

Under salinity conditions, plants show adverse effects in germination, growth, and reproduction that subsequently diminish crop yield [4]. Many modern agricultural crops exhibit a low tolerance to salt, as opposed to wild plants that very often are salt-resistant and can produce significant yields in saline soils. Agricultural production in these salt-affected agricultural environments thus requires the development of salt-tolerant food and fiber crops [5,6]. Additionally, besides crop diversification and the identification of plant-tolerant genotypes, one possible strategy towards crop improvement might be to rely on salt-tolerant root-associated soil microorganisms to augment plant performance and growth under stressful environments [7]. Nowadays there is a growing interest in the identification and use of these microorganisms to promote sustainable crop production under abiotic and biotic stresses [8]. Particularly, arbuscular mycorrhizal fungi (AMF) have been already considered as important bio-ameliorators for saline soils [9,10]. Evelin et al. [1] and Begum et al. [11] report several studies in which an enhanced plant growth and tolerance under salinity stress conditions have been observed for different plant species. In detail, AMF can help the plant to cope with salinity stress by enhancing photosynthetic ability and nutrient uptake and increasing antioxidant defense and osmolyte accumulation. AMF colonize land plants in every ecosystem, even in extreme conditions, such as saline soils [12,13]. Although the excess of salinity can negatively affect AMF [14], some AMF are more stress-tolerant than others and they are usually found in stressed and polluted soils [12,13,15]. Autochthonous AMF ecotypes are, in fact, already adapted to soils with extreme characteristics [16]. The identification of highly efficient native AMF isolates from these unfavorable environments is expected to improve their exploitation in applicative agricultural programs and provide solutions for farmers to improve plant health and productivity under sub-optimal stressful conditions [1,12].

In the present work, soil samples were collected around the roots of three selected halophytes, i.e., *Lycium ruthenicum*, *L. chinense*, and *Suaeda salsa*, in the dry desert region of the Minqin Oasis located in the northwest of China (Gansu province), and their mycorrhizal potential was evaluated by both morphological and molecular analyses. 

## 2. Materials and Methods

### 2.1. Study Site and Soil Sampling

**Figure 1:**
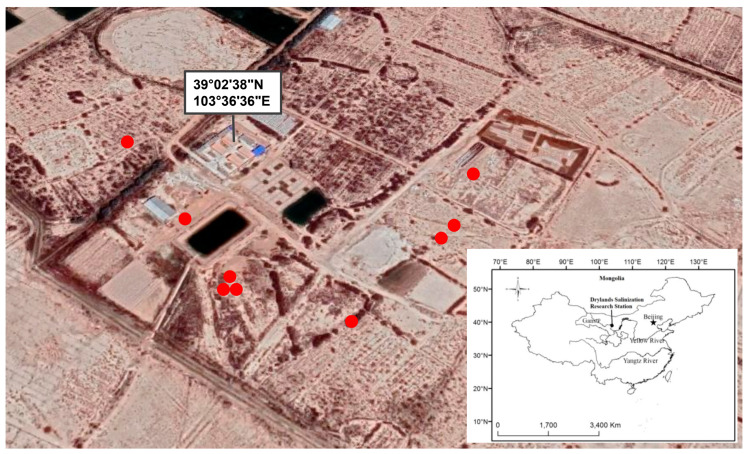
Study area. Red dots represent the sampling points (Landsat imagery, Google Earth).

The study was conducted at Minqin Drylands Salinization Research Station of the Key Laboratory of Deserts and Desertification, Northwest Institute of Eco-Environment and Resources belonging to the Chinese Academy of Science (Figure 1). The station, located in Xiquzhen, Gansu province in China (39°02′ N, 103°36′ E), was established to study the eco-environment of the area with the purpose of evaluating possible solutions against aridity and salinity. The Minqin Oasis, as well as the salinization research station, has a typical arid continental climate [3,17]. Due to the arid climate, agriculture depends heavily on irrigation. Around the station, several species were able to survive the extreme conditions of soil salinity and water scarcity, such as halophytes i.e., *Haloxylon ammodendron*, *Kalidium foliatum*, *Suaeda salsa*, *Nitraria sibirica*, *Peganum nigellastrum*, *Reaumuria songarica*, *Nitraria tangutorum*, *Tamarix hispida*, *Tamarix elongata*, *Lycium ruthenicum*, and *Lycium chinense* (Figure S1). The soil characteristics at a soil depth of 0–20 cm of the study area are reported in Table S1. In Figure 1, the red dots represent the points of sampling carried out in the area around the research station. Samples were taken at the end of the growing season in 2015 (June, summer) because during this period the plants present their full splendor (with flowers). Soil samples were randomly and carefully taken from the root zone, 20 cm deep, of three individuals belonging to the selected halophytes: *Lycium ruthenicum* (S Lr), *L. chinense* (S Lc), and *Suaeda salsa* (S Ss) and were homogenized to be considered as a single sample for each plant species (Figure S1). 

### 2.2. Preparation of Trap Plants and Cultivation

Soil samples were (i) stored at −20 °C, for molecular analyses, and (ii) used to set up a greenhouse experiment with three native halophytes: *L. ruthenicum* (Lr), *L. chinense* (Lc), and *P. nigellastrum* (Pn) as trap plants, due their good germination performance (Figure S1). *P. nigellastrum* was chosen instead of *S. salsa* because of the difficulty of obtaining seedlings of this last species in our growing conditions. In detail, six pots, each with its own pot saucer, were prepared (two for each plant species) using a 50:50 1 mm sieved soil:sterilized sand mixture, to facilitate the drainage, and were irrigated with saline nutritional solution (Hoagland ½) [18] once a week to mirror the native environmental conditions. Trap plants were grown in a greenhouse, separately from the other plants, inside portable and collapsible plant isolators (i.e., rearing cages). 

### 2.3. Morphological Analysis for AMF Detection

After seven months of greenhouse cultivation in pots, *Lycium* spp. and *Peganum* sp. roots were harvested, cleaned and, stained with 0.1% (*w*/*v*) cotton blue in 80% lactic acid overnight, then de-stained three times with lactic acid for 18 h, cut into 1 cm long segments and placed on microscope slides for morphological observations [18]. Root pieces from the same plants were collected and kept in −20 °C for the subsequent molecular analysis. At the same time, the pot substrate (containing soil and sand) were used for spore separation (morphological examination).

### 2.4. Root and Soil DNA Extraction, Nested PCR Amplification, Cloning, and RFLP Typing

To identify the AMF communities associated to the oasis stressed environment we performed a molecular characterization directly on DNA extracted from (1) two replicates of the three soils (S): S Ss (*S. salsa*), S Lr (*L. ruthenicum*), and S Lc (*L. chinense*), and (2) roots (R) of *L. ruthenicum* (R Lr), *L. chinense* (R Lc), and *P. nigellastrum* (R Pn) grown as trap plants in pots. 

To overcome the difficulties of mixed starting material, the species composition of AMF in soil and roots were analyzed by a nested PCR approach. The NS1/NS4 universal primers were used in the first amplification, followed by nested amplification with the AMF-specific primers AML1 and AML2 to amplify a fragment of the SSU rDNA (750 bp) [19]. A negative control was included in the PCR to check for contamination. All PCR products were checked using 1.5% agarose gel stained with ethidium bromide (Merck, Darmstadt, Germany). PCR products were then purified using a Wizard^®^ SV Gel and PCR Clean-Up System (Promega Corporation, Madison, WI, USA) cloned into pGEM-T Easy (Promega, Madison, WI, USA) and transformed into *Escherichia coli* (Xl1 blue). Putative positive transformants were screened in each resulting SSU rRNA gene library, using a second AML1/AML2 amplification following the same PCR conditions described above.

Thirty-two positive clones of the six root clone libraries were tested for restriction fragment length polymorphism (RFLP). RFLP analysis was carried out by independent digestion with HinfI and Hsp92II, according to the manufacturer’s instructions (Promega), and analyzed with 2.5% agarose (in tris-borate-EDTA; TBE) gel electrophoresis. Examples of each RFLP type were chosen for root DNA sequencing, which was performed by Ludwig Maximilians—Universität Munchen (LMU) Sequencing service (Munich, Germany) using the AML2 reverse primer. Sixty-four positive clones for each of the three soil clone libraries were instead sent directly to the LMU sequences service. 

### 2.5. Phylogenetic Analysis

Sequences were edited manually with MEGA 6 (Available online: https://www.megasoftware.net/). Non-AMF sequences were discarded after BLASTN analysis. Environmental sequence affiliation was obtained using the evolutionary placement algorithm (EPA) included in RAxML version 8 (Available online: https://github.com/stamatak/standard-RAxML). The sequences obtained in the present work were aligned with 194 reference sequences, selected to represent the main taxonomic groups in the Glomeromycota, and the 18S ribosomal RNA sequence of *Mortierella polycephala* (X89436). Due to the most recent updates in taxonomy and using the 18S-ITS-28S regions or the 28S region alone, sequences for several taxa were not available. The alignment was carried out with MAFFT 7 using the “auto” option (Available online: http://mafft.cbrc.jp/alignment/server/). The alignment and a reference tree were provided as input for the RAxML–EPA analysis. For each sequence, the EPA assigns a place in the phylogenetic tree used as backbone (Figure S2). The reference tree used in the RAxML–EPA analysis was built with the 195 sequences mentioned above. Reference sequences were aligned with MAFFT 7 using the “auto” option. To increase the accuracy of the phylogenetic reconstruction, indels coded by FastGap v1.2 (Available online: http://www.aubot.dk/FastGap home.htm) were added to the nucleotide alignment, as described in [20]. A maximum likelihood (ML) phylogenetic inference of the partitioned alignment was computed through the CIPRES web portal [21] with RAxML ver. 8.2.12 [22], choosing GTRGAMMA as a substitution model and 1000 iterations for rapid bootstrapping. A *Mortierella polycephala* sequence (X89436) was used to root the tree. Sequences representative for each phylogenetic node were deposited in the NCBI database (MN597010- MN597041).

## 3. Results and Discussion

**Figure 2:**
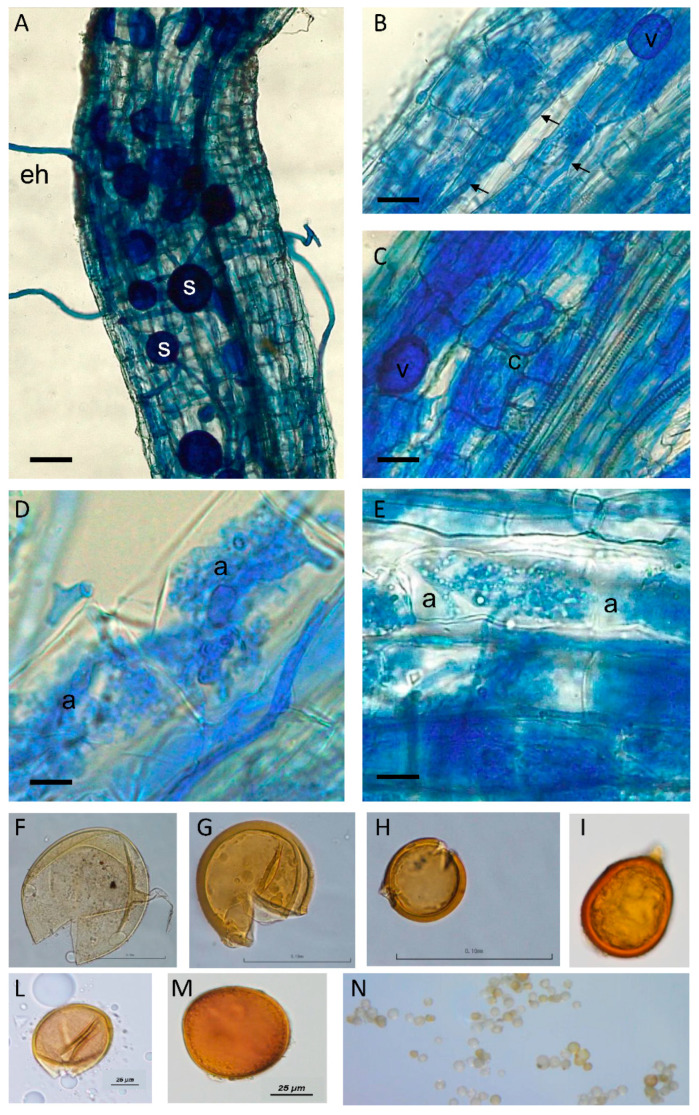
Arbuscular Mycorrhizal Fungal (AMF) colonized *Lycium ruthenicum* roots after staining with cotton blue to highlight several fungal structures. (**A**) External hyphae (eh) and spores (s) are evident around the root. Bar = 200 μm. (**B**) At a higher magnification, intercellular hyphae are evident (arrows). A vesicle (v, a storage structure) is also evident. Bar = 150 μm. (**C**) A coil (c) is present inside a cortical cell, as well as a vesicle (v). Bar = 115 μm. (**D**,**E**) Magnification of cortical cells containing arbuscules (a), which are considered the key symbiosis structures where nutritional exchanges occur. Bars = 50 and 30 μm, respectively. (**F**–**N**) Spores of different AMF morphotypes, mirrored by the different spore morphology, isolated from the soil of trap plants.

Despite the low mycorrhizal affinity of the halophytes that has been reported [23], the evaluation of root colonization allowed the verification of the presence of typical AMF structures (arbuscules, hyphae, and vesicles) inside the roots of the considered trap plants, i.e., two *Lycium* species and *P. nigellastrum* (Figure 2 for *L. ruthenicum* and not shown for the other two plant species). These results suggested the presence of AMF propagules in the original collected soils and their germination capability and infection. This statement has also been confirmed by spore extraction and microscopical observations, showing the presence of spores with different morphologies typical of different AMF taxa (Figure 2). 

The density of AMF spores in desert saline areas has been controversially reported, i.e., very low, with a general inhibition of germination and AMF hyphal growth [24], or not significantly decreased with a relatively high spore number (mean of 100 per 10 g soil) [25]. This fact has been partially explained by AMF low root colonization levels that might conversely stimulate spore production under severe saline conditions [26]. As reported by Antunes et al. [27], different AMF behaviors might result from adaptations towards environmental conditions, prevailing at their place of origin, and by differences in AMF structural traits. Such well-adapted AMF ecotypes may then have unique physiological capabilities to cope with the extreme conditions prevailing in the desert, such as long drought seasons leading to desiccation, and high salt concentration.

Trap plants have been previously used by Ferrol and colleagues [28] to bait the whole population of native AMF, since AMF spores in soil samples taken directly from the ecosystem of *Pistacea lentiscus*, one of the most representative shrub species from Mediterranean regions and a target plant for revegetation programs, was relatively low, and the only AMF species recognized in the native soil was *Paraglomus occultum*. In our work, the DNA extracted from trap plant roots, as well as from soils, was successfully amplified using the nested PCR approach, obtaining a fragment of the expected size (ca. 750 bp). Overall, 288 clones from roots and soils were sent for sequencing and 199 (60 from root samples and 139 from soils) were available for phylogenetic analysis (Table 1; Figure S3). Phylogenetic analyses revealed AMF belonging to two out of four orders of the phylum Glomeromycota: sequences were distributed over four families (*Glomeraceae*, *Claroideoglomeraceae*, *Diversisporaceae*, and *Gigasporaceae*) and different clades. In total, 26 AMF taxa were retrieved: nine AMF taxa from rhizosphere soils of *L. ruthenicum*, *L. chinense*, and *Suaeda salsa* (Gi1, Sept2, Glo4, Do1, Do2, Rh1, Rh2, Glo6, Glo7) and 17 AMF taxa from roots of *L. ruthenicum* (Lr), *L. chinense* (Lc), and *Peganum nigellastrum* (Pn) (Div1, Div2, Div3, Div4, Cla1, Cla2, Cla3, Cla4, Cla5, Fun1, Fun2, Fun3, Sept2, Glo1, Glo2,Glo3, Glo5) (Table 1). The most abundant genera retrieved from roots were represented by *Diversispora* accounting for 38.6% of the total clones analyzed by RFLPs, followed by *Claroidoglomus*, and *Glomus*, representing 27.2% and 24.4%, respectively. The less abundant taxa retrieved from roots were those belonging to *Funneliformis* (2.8%) and *Septoglomus* genera, accounting for 2.8% and 2.7% of the total clones, respectively.

Despite the AMF primer specificity, 4.3% of the total clones were referred to as contaminants (plants, other fungi), as already reported in literature [29]. Each trap plant species captured a different plethora of AMF taxa. In the roots of *L. ruthenicum* (Lr), the most abundant taxa were those ascribed to *Claroidoglomus* and *Diversispora*, while on the contrary, *L. chinense* (Lc) was mainly colonized by many different taxa affiliated to Glomeraceae (i.e., *Glomus* and *Funneliformis* spp.), while in the roots of *P. nigellastrum* (Pn), AMF belonging to different *Diversispora* phylogroups and, to a lesser extent, *Claroidoglomus* and *Septoglomus* taxa were retrieved. From soil-based sequencing, most sequences were ascribed to *Glomeraceae* and, in particular, to taxa included in the basal *Rhizophagus–Sclerocystis–Ohelia* groups (26.6%), followed by *Rhizophagus/Rhizoglomus* taxa (i.e., *Rh. intraradices*, *Rh. arabicus*), representing 20.8%, *Septoglomus* (19.4%), and taxa belonging to *Dominikia* and *Dominikia*-related phylogroups (14.3%). In addition, 17.2% of sequences were assigned to *Gigasporaceae* (*Gi. margarita*) and only 0.7% to *Claroidoglomus*. 

Our results reveal that the AMF communities identified were only partially shared by the three plant species studied, as expected. Each trap plant species captured a different plethora of AMF taxa, suggesting that the vegetation in the considered stressed area, including *Chenopodiaceae* spp. (often described as not mycorrhizal plants), retains potential rhizospheric communities, offering a natural wealth of AMF and probably other root-associated microorganisms (e.g., plant growth-promoting bacteria), as reported in different ecosystems. These results are intriguing and confirmed previous studies where rhizospheric soil from *Chenopodiaceae* plant species, such as *Sueda* spp., maintained AMF potential at the same levels of other mycorrhizal plant host species in saline-stressed semi-desert and desert environments [26,30]. Both soil-based and plant root sequencing showed AMF taxa corresponded to isolates already reported in similar stressed environments, either in China [31,32] or the Mediterranean basin, where many ecosystems are subjected to desertification and a rapid increase in soil salinity [13,28,33]. In detail, our findings confirmed AMF species belonging to Glomerales, such as *Rh. intraradices*, *Fu. mosseae*, *Fu. geosporus*, *Cl. etunicatum*, *Cl. claroideum*, *Se. constrictum*, as the most frequently retrieved taxa in saline and drought environments ([16] and references therein). The presence of species belonging to the *Gigaspora* genus was also previously found in ecosystems without anthropogenic disturbances [34].

Overall, our results provide new information useful for the optimization of AMF application to sustain agriculture in saline environments, promoting the selection and the use of AMF associated with native plants. Native AMF consortia, consisting of several taxa already adapted to extreme conditions and with different symbiotic behaviors, can, in fact, be more efficient at preserving these ecosystems from advancing desertification and secondary soil salinization due to irrigation. More specifically, AMF can mitigate the vulnerability of horticultural crops to water scarcity and soil salinity, thus allowing viable cultivation in extreme environments. Moreover, digging into the functional diversity of autochthonous AMF ecotypes for their impact on plant tolerance to environmental stresses, will promote a most effective use of these beneficial soil microorganisms in sustainable agriculture programs in a climate change scenario.

## Figures and Tables

**Table 1:** Phylotypes of arbuscular mycorrhizal fungi, identified using the AML1–AML2 primer pair, in rhizosphere soils of *L. ruthenicum*, *L. chinense*, and *S. salsa* and in roots of the trap plant species *L. ruthenicum*, *L. chinense*, and *P. nigellastrum.*

Reference AMF	Node	Confidence Values	Phylogroups	N° of Clones Sequenced	
				Soil	Root
*Gigaspora margarita*	I111	1.00	Gi1	24	0
*Diversispora spurca*	I182	0.80	Div1	0	1
*Diversispora spurca*	I183	0.83	Div2	0	7
*Diversispora* sp.	I206	0.74	Div3	0	1
*Diversisporaceae* sp.	I222	0.95	Div4	0	15
*Claroideoglomus* sp.	I236	1	Cla1	1	1
*Claroideoglomus* sp.	I240	0.95	Cla2	0	1
*Claroideoglomus* sp.	I239	0.36	Cla3	0	6
*Claroideoglomus* sp.	I267	0.35	Cla4	0	2
*Claroideoglomus* sp.	I268	0.83	Cla5	0	3
*Funneliformis* sp.	I281	0.82	Fun1	0	1
*Funneliformis mosseae*	I287	0.95	Fun2	0	1
*Funneliformis* sp.	I291	0.78	Fun3	0	2
*Septoglomus* sp.	I304	0.90	Sept1	1	0
*Septoglomus* sp.	I297	1.00	Sept2	26	3
*Glomeraceae* sp.	I312	1.00	Glo1	2	2
*Dominikia iranica*	I321	0.68	Do1	1	0
*Glomeraceae* sp.	I322	1.00	Glo2	3	4
*Glomeraceae* sp.	I326	0.93	Glo3	8	3
*Glomeraceae* sp.	I327	0.95	Glo4	5	0
*Dominikia indica*	I339	1.00	Do2	1	0
*Glomeraceae* sp.	I340	1.00	Glo5	11	7
*Rhizophagus intraradices*	I351	0.80	Rh1	1	0
*Rhizophagus arabicus*	I373	0.89	Rh2	28	0
*Glomeraceae* sp.	I380	0.85	Glo6	26	0
*Glomeraceae* sp.	I383	1	Glo7	1	0
**Total**			**26**	**139**	**60**

## Supplementary Materials

Supplementary materials can be found at https://www.mdpi.com/2309-608X/6/2/80/s1. Figure S1: Halophytic species present in the study area: (**A**) *Haloxylon ammodendron*, (**B**) *Kalidium foliatum*, (**C**) *Lycium chinense*, (**D**) *Lycium ruthenicum*, (**E**) *Nitraria sibirica*, (**F**) *Nitraria tangutorum*, (**G**) *Peganum nigellastrum*, (**H**) *Reaumuria songarica*, (**I**) *Suaeda salsa*, (**J**) *Tamarix elongata*, and (**K**) *Tamarix hispida*. Figure S2: Phylogenetic backbone tree. A 50% majority rule consensus phylogram produced from a maximum likelihood analysis of 194 partial SSU rDNA reference sequences, selected to represent the main taxonomic groups in the Glomeromycota. A sequence of *Mortierella polycephala* (X89436) was added as an outgroup. The reference tree served as base for the one-by-one insertion, and therefore affiliation, of the query sequences. Figure S3: Node tree. Position of the nodes on the reference tree after the EPA analysis. The tree is shown as a cladogram to ease the visualization of the nodes, highlighted in yellow, where the query sequences were placed.
