# Supplementary material for: Native Arbuscular Mycorrhizal Fungi Characterization from Saline Lands in Arid Oases, Northwest China
Source: J Fungi (Basel). 2020 Jun 6;6(2):80. doi: 10.3390/jof6020080 (PMC7344694; doi:10.3390/jof6020080)
Supplement: Supplementary file 1 [file jof-06-00080-s001.zip › SM/Supplementary Table 1_soil characteristic.docx]

**Supplementary Table 1**: Soil characteristics at soil depth 0-20 cm of the study area.

| Parameter | Soil layer  (0-20 cm) |
| --- | --- |
| Clay (%) | 7.00 |
| Silt (%) | 44.3 |
| Sand (%) | 48.7 |
| Bulk density (g cm^-3^) | 1.40 |
| pH | 7.5 |
| ECe (dS m^-1^) | 2.0 |
| Ca ^2+^ (g kg^-1^) | 0.053 |
| Mg ^2+^ (g kg^-1^) | 0.031 |
| K ^+^ (g kg^-1^) | 0.016 |
| Na ^+^(g kg^-1^) | 0.032 |
| N total (g kg^-1^) | 0.757 |
| Total carbon (g kg^-1^) | 17.3 |
| Available P (mg kg^-1^) | 15.58 |
|  |  |
