# Supplementary figures and images for: Native Arbuscular Mycorrhizal Fungi Characterization from Saline Lands in Arid Oases, Northwest China
Source: J Fungi (Basel). 2020 Jun 6;6(2):80. doi: 10.3390/jof6020080 (PMC7344694; doi:10.3390/jof6020080)

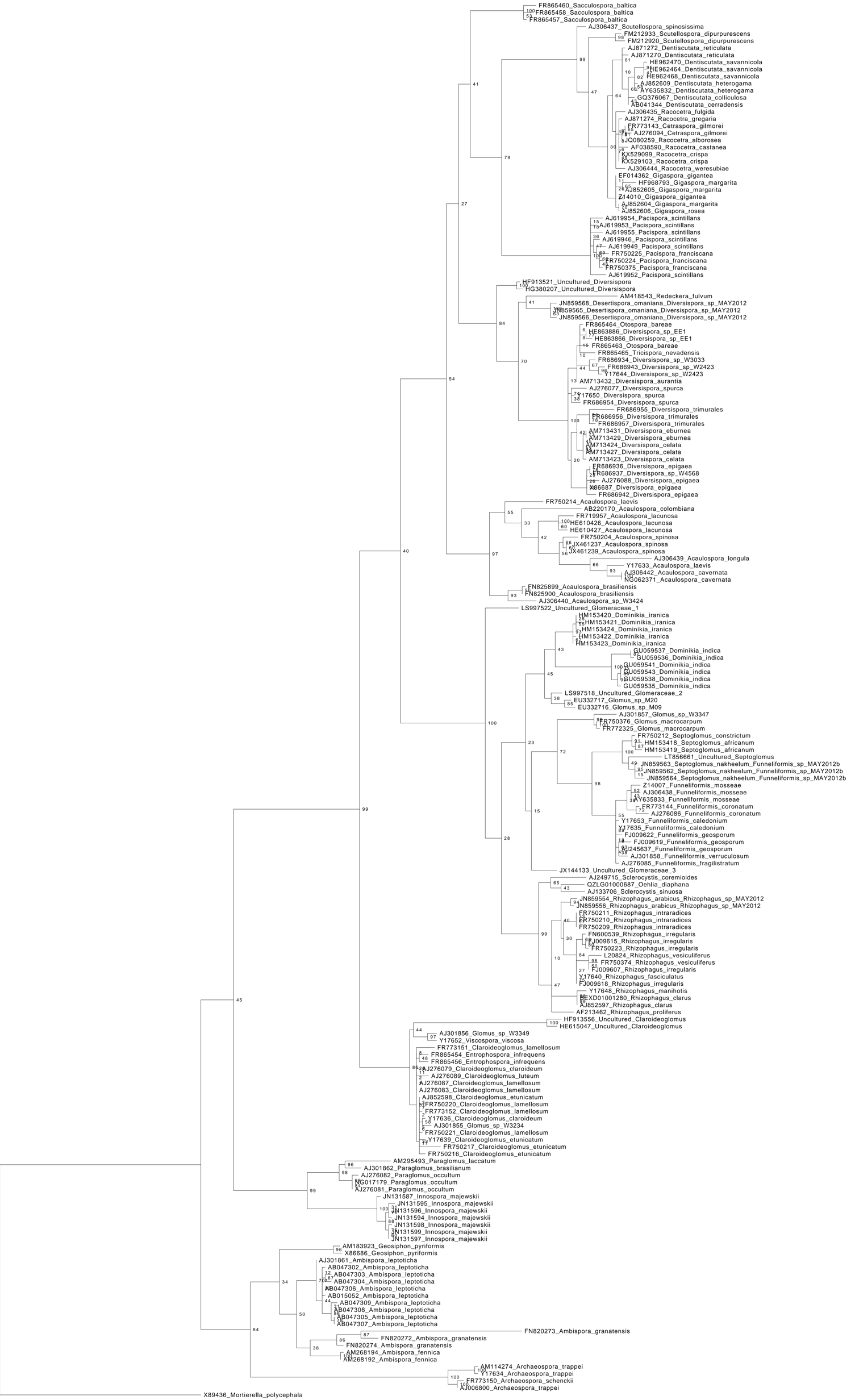

Supplement: Supplementary file 1 [file jof-06-00080-s001.zip › SM/Fig S2_ref_tree_result.pdf]

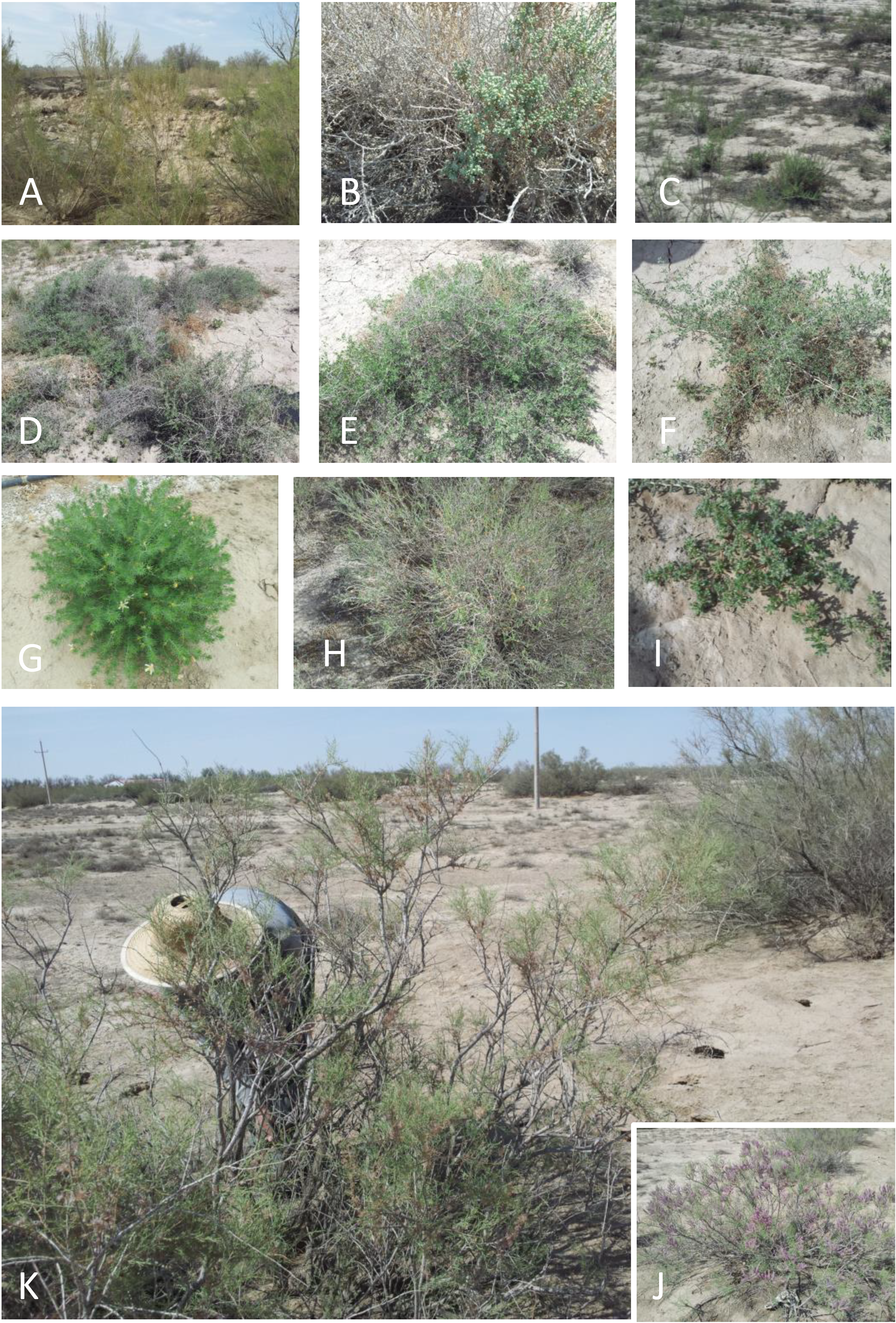

Supplement: Supplementary file 1 [file jof-06-00080-s001.zip › SM/Figure_S1.tif]

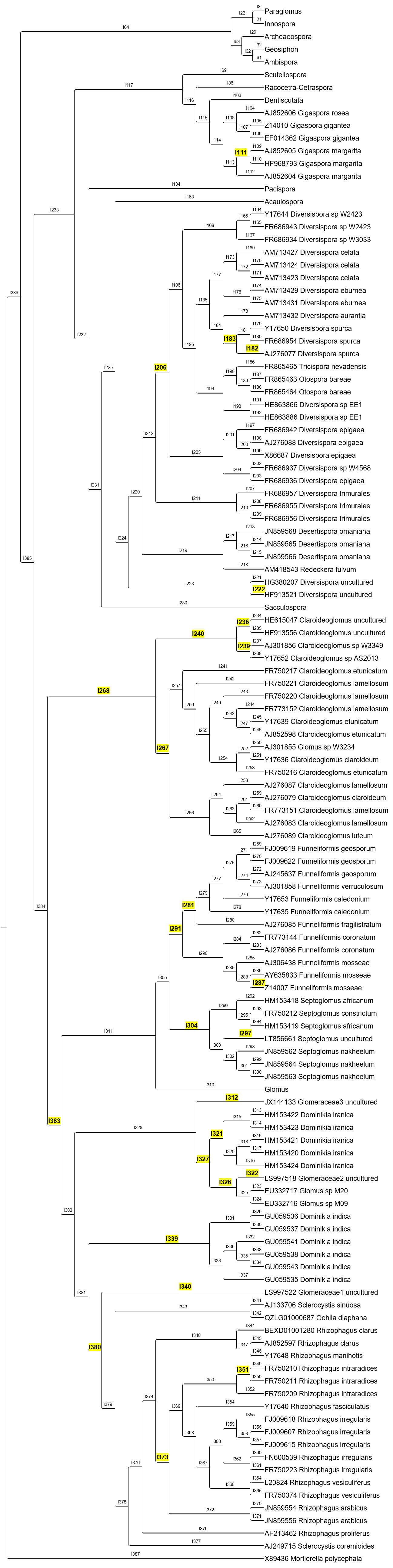

Supplement: Supplementary file 1 [file jof-06-00080-s001.zip › SM/Figure_S3.jpg]
